# Editorial: Plasmodesmata: Recent Progress and New Insights

**DOI:** 10.3389/fpls.2022.840821

**Published:** 2022-03-24

**Authors:** Tessa Burch-Smith, Manfred Heinlein, Jung-Youn Lee

**Affiliations:** ^1^Donald Danforth Plant Science Center, St. Louis, MO, United States; ^2^Institut de Biologie Moléculaire des Plantes, CNRS, Université de Strasbourg, Strasbourg, France; ^3^Department of Plant and Soil Sciences, Delaware Biotechnology Institute, University of Delaware, Newark, DE, United States

**Keywords:** plasmodesmata, cell-to-cell movement, biotechnology, plant-pathogen interactions, symplasmic domains, cytoskeleton

In this Frontiers Research Topic, readers will find a collection of research articles, mini-reviews, and opinion papers that focus on new findings and progress regarding plasmodesmata in the context of plant development and plant-pathogen interactions. Specifically, several reports present findings related to the targeted trafficking of endogenous and pathogen-derived proteins to or through plasmodesmata, or the role and regulation of plasmodesmata in defining symplasmic domains. The collection also includes articles that review progress with respect to cytoskeletal connections to basic plasmodesmal function or to interspecific plasmodesmata formed between hosts and their parasitic plants, or share perspectives on how plasmodesmal research may be relevant to addressing critical issues in producing resilient crops in the face of imminent challenges associated with climate change.

In higher plants, virtually all sister cells are connected to each other *via* the primary plasmodesmata formed at the division wall during cell division. However, as cells grow and differentiate, those plasmodesmata can undergo temporary closing or various structural modifications such as those that lead to the formation of secondary/modified plasmodesmata or to disconnection by severing or complete disintegration. These events sometimes lead to the symplasmic isolation of cells. Voitsekhovskaja et al. investigate how secondary plasmodesmata may differentially form depending on how they load sugar into the phloem, i.e., using an apoplastic or symplastic path. This study reveals that secondary plasmodesmata formation is enhanced in symplastic loaders, particularly at the cell walls joining epidermal cells and epidermal with mesophyll cells. In addition, comparative analysis of carbohydrate composition suggests that secondary plasmodesmata formed between the two cell layers are likely used to traffic photosynthetic assimilates. Collectively, these findings raise the intriguing possibility that the epidermis and mesophyll could together comprise a symplastic domain in symplastic loaders. Godel-Jedrychowska et al. investigate how symplamic domains are formed in zygotic and somatic embryos during their development. Their study suggests that although the symplasmic domains form similarly in both types of embryos, there are a few qualitative differences such as the timing of establishing domain boundaries and the size of molecules that can move between cells. Krause group addresses the functional specialization of secondary plasmodesmata (Fischer et al.), examining what is known about interspecific plasmodesmata formed between parasitic plants and their plant hosts and provides cogent arguments for the value of parasitic plant-host systems in investigating various aspects of plasmodesmal formation and structure, and the establishment of symplastic domains.

Two reports describe findings about plasmodesmata in the context of plant development, one related to the role of cytokinin in plasmodesmal function and the other to transcription factor movement critical for xylem development. Various reports have shown that plant hormones, such as auxin, abscisic acid, gibberellin, and salicylic acid, regulate plasmodesmal status, and/or vice versa. Adding to the list of hormones linked to plasmodesmal function, Horner and Brunkard show that direct application of a cytokinin, *trans*-Zeatin, or virus-induced gene silencing of the components of the cytokinin signaling pathway both bring about changes in plasmodesmal permeability. The transcription factor AT-HOOK MOTIF NUCLEAR LOCALIZED PROTEIN(AHL)4 is a mobile member of a large protein family, which is necessary for the proper xylem differentiation in Arabidopsis. Using domain swapping between AHL4 and a non-mobile member, AHL1, followed by genetic analyses, Seo and Lee now show that a specific C-terminal domain in AHL4 determines the mobility of the protein, and that AHL4 mobility from the stele to the endodermis and xylem precursor cells is vital for xylem development.

Chritiaan van der Schoot and his team examine the relationship between lipid bodies and plasmodesmata in the shoot apical meristem in hybrid aspen and analyze the proteins associated with lipid bodies in dormant buds (Veerabagu et al.). Their findings indicate how lipid bodies may function as a putative delivery system for plasmodesmal proteins along the actin cytoskeleton to plasmodesmata. A mini review summarizes the association of actin with plasmodesmata (Diao and Huang) focusing on class I formins, actin-binding proteins involved in actin polymerization. Several class I formins localize to plasmodesmata including AtFH1 and AtFH2, which are required to maintain plasmodesmal permeability.

Reflecting recent interest in the role of plasmodesmata as the battleground against microbial intruders, more proteins encoded by various microbial pathogens are identified to target plasmodesmata. Kyaw Aung's team presents evidence showing that bacterial effector proteins can traffic between cells (Li et al.), adding to the previous findings from fungal and oomycete systems (Cheval and Faulkner, 2018; Iswanto et al., 2021). They show that the effector movement is restricted by accumulation of callose at plasmodesmata and that an effector targeted to the plasma membrane is more efficiently able to move between cells than a mutant version that does not associate with the plasma membrane. How plasma membrane association may facilitate the protein's intercellular movement and how broadly this putative mechanism may apply are interesting questions for future investigations. In addition, it would not be surprising if beneficial bacteria also deploy effectors to bring about potential non-cell-autonomous effects.

Notably, three research groups review and discuss potential applications of plasmodesmal research to improve crop health and yield. As the effects of global climate change become more pronounced in the coming years, there is no doubt that a variety of biotechnological approaches will be needed to enhance crop adaption. Along this line, Liu et al. succinctly summarize a large body of research on the ways pathogens may manipulate plasmodesmata to facilitate infection and how plants can deploy plasmodesmata-centered defenses to limit infection. Possible strategies of engineering plasmodesmata to enhance defense responses, for example by targeting callose metabolizing enzymes are also discussed. Iswanto et al. discuss plasmodesmal proteins involved in abiotic stress and in host-pathogen interactions as potential targets for gene editing using CRISPR/CAS9 technologies. The urgency to consider the importance of plasmodesmata research for crop improvement is furthermore underscored in the Perspective article from the Heinlein lab (Amari et al.). It highlights the potential impact of global warming on virus propagation in infected plants and agricultural productivity and collates work spanning decades that clearly indicates the increased susceptibility of plants to viral cell-to-cell movement at higher temperatures. Perhaps, the regulation of plasmodesmata may hold a promise as a new target for crop engineering and the time may be ripe for that exploration.

## Author Contributions

TB-S wrote the first draft of the editorial. J-YL revised the draft and added additional sections, and MH edited. All authors contributed to the conception and solicitation of this Research Topic.

## Funding

This work was partially supported with funding provided by the National Science Foundation (MCB1820103 to J-YL and MCB 1846245 to TB-S) and the Agence Nationale de la Recherche (ANR-21-SUSC-0003-01 to MH).

## Conflict of Interest

The authors declare that the research was conducted in the absence of any commercial or financial relationships that could be construed as a potential conflict of interest.

## Publisher's Note

All claims expressed in this article are solely those of the authors and do not necessarily represent those of their affiliated organizations, or those of the publisher, the editors and the reviewers. Any product that may be evaluated in this article, or claim that may be made by its manufacturer, is not guaranteed or endorsed by the publisher.
